# Correction

**DOI:** 10.1080/23335432.2025.2576271

**Published:** 2025-10-23

**Article title**: Assessing single camera markerless motion capture with OpenSim inverse kinematics during upper limb activities of daily living

**Authors**: Bradley Scott, Mhairi McInnes, Edward K. Chadwick, & Dimitra Blana

**Journal**: *International Biomechanics*

**Bibliometrics**: Volume 12, Number 01, pages 35-47

**DOI**: https://doi.org/10.1080/23335432.2025.2556187

**Article title**: Experimental evaluation of hard hat performance during impacts to a forward-flexed head

**Authors**: Arthur Santos, James Sorce, Mohammadhadi Masoudi, Alexandra Schonning, & Grant Bevill

**Journal**: *International Biomechanics*

**Bibliometrics**: Volume 12, Number 01, pages 48-57

**DOI**: https://doi.org/10.1080/23335432.2025.2560410

**Article title**: Biomechanical comparison of two tension band wiring techniques for transverse patella fracture fixation: an in vitro study of 3-D printing models

**Authors**: David Sáez Martínez, Juan Carlos del Real Romero, Emilio Calvo Crespo, & Felipe López-Oliva Muñoz

**Journal**: *International Biomechanics*

**Bibliometrics**: Volume 12, Number 01, pages 58-65

**DOI**: https://doi.org/10.1080/23335432.2025.2562270

When the above articles were first published, there were errors in the pagination of the articles. These errors have now been corrected online.

Taylor & Francis apologizes for this error.

